# The Challenges of Canadian Pharmacare Are More Complicated Than Acknowledged Comment on "Universal Pharmacare in Canada"

**DOI:** 10.15171/ijhpm.2020.20

**Published:** 2020-02-15

**Authors:** Kristina M. L. Acri née Lybecker

**Affiliations:** Department of Economics and Business, Colorado College, Colorado Springs, CO, USA.

**Keywords:** Universal Pharmacare, Health Policy, Equity, Canada, Pharmaceuticals

## Abstract

This commentary considers two editorial pieces, written by Hajizadeh and Edmonds, and Lewis, which address universal pharmacare in Canada. The pieces focus on the social inequities of the existing system and the challenges of successful implementation. After identifying the significant strengths of both articles, this commentary then delves into the reasons why universal pharmacare may not be the solution, and identifies numerous thorny issues that will complicate the implementation of such a publicly funded program. Both discussions point to the need for caution and transparency going forward.

In response to an invitation from the editors, it is my pleasure to provide this commentary on two pieces on Universal Pharmacare in Canada that recently appeared in this journal.^1,2^ The first editorial, by Hajizadeh and Edmonds, focuses on the social inequities in the burden of catastrophic out-of-pocket expenses on drugs and pharmaceutical products (COPEDP) and the ways in which universal pharmacare would reduce COPEDP and promote greater equity in the Canadian healthcare system. The second piece, a perspective by Lewis, identifies five constituencies that must be involved in the development of a national program, and describes the conditions under which a genuinely fair, effective and efficient universal pharmacare plan can be developed for Canada.

For some perspective, consider that in a comparison of combined public and private spending on pharmaceuticals, spending per capita in Canada exceeds all other Organisation for Economic Co-operation and Development (OECD) countries apart from the United States and Switzerland.^3,4^ In addition, some analysts claim that Canada pays more while providing less access.^5^ Understandably, this has spurred proposals for a Canadian pharmacare program, in the hopes that it would improve access and reduce pharmaceutical expenditures. While claims of inadequate coverage are debatable, they contribute to the public perception that a national pharmacare program is a necessary next step. The Hoskins Report (*A Prescription for Canada: Achieving Pharmacare for All*^6^) addresses this concern, proposing how Canada may achieve universal drug coverage.

Hajizadeh and Edmonds effectively make the point that COPEDP disproportionally impact low-income households, seniors and households using social assistance, creating financial hardship for many of these individuals. These findings echo multiple earlier studies.^7,8^ The article provides clear evidence of social inequities, illustrated with numerous charts, graphs, and empirical data from Statistics Canada (2010-2015). The result is convincing evidence that individuals of the lowest socio-economic status suffer from substantial variation in COPEDP across provinces, a burden that higher-income households never face.

In the second editorial, Lewis identifies the challenges, in great detail, of successful implementation through the lens of five major political constituencies: physicians, the retail pharmacy business, private drug insurers, the pharmaceutical industry and the public. In this brief piece, the author astutely pinpoints several of the most significant challenges of implementation: addressing the free market commodification of prescription medicines, the necessity of making compromises to keep marginal drugs out of the formulary, and the need to commit to the greatest good for the greatest number. Some of these issues may be addressed through the promotion of evidence-based practice and prudent use, but it will be essential to say “no” to some constituencies, which will undoubtedly draw criticism.

Notably, Lewis identifies pharmacare as “medicare’s Achilles heel and unfinished business,” a description that is both familiar and somewhat misleading. Notably, as detailed by Barua the assertion that Canada is the only industrialized country with a universal healthcare system that does not provide national drug coverage to its citizens is entirely false.^9^ In actuality, Canada is the only country in the industrialized world with universal healthcare that does not have a second, private tier of healthcare. This is significant in that public drug coverage is affordable to the governments in most other countries due to the savings achieved by shifting part of the burden of paying for healthcare to the private sector. Consider, at the extreme, the case of the United States. The free market pricing of drugs to the private sector allows for government negotiations for lower prices in the public sector. It is arguable that the free market prices paid by US consumers subsidize pharmaceutical research and development costs for the rest of the world since all other countries rely on some type of price controls or negotiated discounts. Thus, the premise of comparing Canada to its international peers is misleading and inappropriate.

Both articles identify the lack of coverage for prescription drugs as a real and concerning problem, but – as always – the devil is in the details. The articles make the point that fair, effective, efficient and equitable healthcare coverage is needed in Canada. This applies to both existing coverage and any pharmacare coverage that may be in the works. In addition, both articles identify implementation of universal pharmacare as both a formidable challenge and as a potential step toward addressing the need for healthcare equity in Canada.

In reflecting on these, and other, commentaries on universal pharmacare, it is essential to address several thorny issues head on: social inequity, performance, and the unintended consequences of a publicly funded program. First, the social inequity of the uninsured costs of pharmaceutical drugs must be addressed. These two articles both raise this concern, thus joining a larger body of work that suggests there is evidence that some Canadians may be struggling to cover the costs of their prescription medicines.^10-12^ However, they fail to point out that, under existing programs, lower-income Canadians already have access to some form of provincial insurance that limits out-of-pocket costs for prescription drugs to a minimal share of income, if not more extensive coverage, in every Canadian province.^13^ These articles, and the public discourse in general, lack a clear understanding of the coverage that is already available to the most vulnerable subsets of the Canadian population who may be at higher risk of foregoing their prescriptions due to cost: those with lower incomes (including seniors), the disabled, and patients with chronic medical conditions.

While there is significant evidence of social inequities, there is no evidence that a federal universal pharmacare program would correct the inequity. Considering the performance of the publicly funded healthcare system, there are good reasons to be cautious. According to a 2018 report, “although Canada’s is among the most expensive universal-access healthcare systems in the OECD, its performance is modest to poor.”^5^ Specifically, there is an imbalance between the benefits Canadians receive relative to the cost of their healthcare system. In comparing universal-access healthcare systems in the OECD, Canada’s performance for availability and access to resources is generally below that of the average OECD country, and an evaluation of Canada’s use of resources and quality and clinical performance is mixed. Is there reason to believe that a universal pharmacare program would perform any better?

Again, the articles neglect to describe the coverage that is already available to Canadians with lower incomes in order to ensure they have access to necessary prescription drugs. Understanding this aspect of current health policy is critical to the debate surrounding how well lower income Canadians are already protected from the potentially high costs of prescription medicines. In an excellent review of the issue, Esmail provides an overview of drug insurance coverage for low income Canadians across Canada, including the definition of “low income” in each province.^14^ The study finds, “A review of provincial drug insurance coverage reveals that lower income Canadians have access to at least catastrophic insurance (limiting out-of-pocket costs to a small percentage of income) for prescription drugs, if not more extensive coverage, in every Canadian province. Coverage for lower income children and seniors tends to be relatively more generous than for non-senior adults, particularly those without children. Recipients of social assistance have coverage at very low or no premiums, deductibles, and co-payments in every province.” While this coverage is important, it is essential to recognize that the intended coverage is not always easy to obtain due to administrative requirements that can make it difficult for individuals with literacy, mobility and other negative social determinants, as well as their healthcare providers. These limitations must be acknowledged^[1]^.

In addition, in their reviews of universal pharmacare, Lewis and Hajizadeh & Edmonds fail to point out that it may not necessarily be the answer and other approaches may offer a better path forward. My research on the experiences of other nations sheds some light on what may be expected from a universal pharmacare program.^15^ Unfortunately, it may not deliver the anticipated benefits. Through an examination of the experiences of New Zealand, Australia, and the United Kingdom, my research points to the difficult decisions that accompany a national publicly funded pharmacare scheme, and the consequences of such programs for patients, physicians, innovators, and the industry. In particular. Universal pharmacare may result in more limited access to new drugs, poorer healthcare outcomes, excess burdens of taxation, and reduced pharmaceutical innovation. While not guaranteed, these consequences are typical of such policies.

As with many public policy proposals, the consequences of a universal, publicly funded pharmacare program must be critically evaluated and properly costed in order to determine whether this policy would benefit Canada and Canadian patients. There are a number of potentially very detrimental consequences and policy-makers should have answers for how they will be avoided or remedied. First, the true tax burden should be calculated and transparently presented. Second, it must be recognized that drug shortages and reduced access may result from such a policy, a consequence that will certainly create hardship for some patients. There is also substantial evidence indicating that lower revenues and profits will reduce pharmaceutical R&D spending and innovation. Finally, it is essential to acknowledge the potential for worsening health outcomes and suboptimal therapeutic substitution.

While this commentary does not embrace universal pharmacare with the same optimism and enthusiasm that Lewis and Hajizadeh & Edmonds do, all three pieces clearly recognize that implementation of such a policy will be difficult and point out that the full spectrum of challenges are not yet fully known. Accordingly, Canada must cautiously approach any policy change that puts patients, innovation, and innovative industries at risk.

## Ethical issues

Not applicable.

## Competing interests

Author declares that she has no competing interests.

## Author’s contribution

KMLAnL is the single author of the paper.

## Endnote

[1] The author is grateful to an anonymous reviewer for making this point and requesting its inclusion in this commentary.

